# Optimized protocol for single-cell and bulk transcriptomic profiling of stabilized hemocytes from *Anopheles gambiae* mosquitoes

**DOI:** 10.1186/s44330-026-00092-5

**Published:** 2026-11-11

**Authors:** Banhisikha Saha, Colton M. McNinch, Carolina Barillas-Mury

**Affiliations:** 1 Laboratory of Malaria and Vector Research, National Institute of Allergy and Infectious Diseases, National Institutes of Health, Rockville, USA; 2 Bioinformatics and Computational Biosciences Branch, Office of Cyber Infrastructure and Computational Biology, National Institute of Allergy and Infectious Diseases (NIAID), National Institutes of Health, Bethesda, MD 20892 USA

**Keywords:** *Anopheles gambiae*, Hemocytes, Single-cell, Bulk RNA-seq, RNA stabilization

## Abstract

**Background:**

Insect hemocytes play a crucial role in the innate immune response against pathogens. However, transcriptomic profiling and analysis of hemocytes in insect vectors, such as *Anopheles gambiae* mosquitoes, are challenging due to their small size, low RNA content, limited numbers, and highly reactive nature.

**Methods:**

To address these challenges, we developed a protocol for single-cell and bulk RNA sequencing of stabilized hemocytes from adult female *An. gambiae* mosquitoes. Immediate stabilization of hemocytes following perfusion prevents transcriptional changes during sample collection and RNA degradation, thereby minimizing technical artifacts. After stabilization, hemocytes are filtered to remove contaminating tissue and ambient RNA introduced by tissue injury during sample collection, enriching for hemocyte-specific transcripts. The stabilized hemocytes can be stored for up to four weeks, allowing for convenient sample storage, transportation, and simultaneous processing of multiple samples, which reduces costs and minimizes batch effects.

**Discussion:**

Our single-cell RNA sequencing stabilization and processing protocol is compatible with the 10X Genomics platform, yielding high-quality and reproducible data. We demonstrate higher hemocyte recovery compared to other contemporary methods, enabling the identification of rare hemocyte populations. However, this protocol requires further optimization to increase the recovery of small-sized prohemocytes (< 5 μm in diameter). Our bulk mRNA stabilization and hemocyte enrichment protocol yields high-quality, intact RNA, and Illumina bulk mRNA sequencing libraries enable an in-depth investigation of gene expression changes in hemocytes. These protocols significantly improve transcriptomic analysis of hemocytes, which is critical for advancing discoveries in vector biology and disease control. Moreover, the protocol can be applied to other mosquito species and likely many other insects with only minor optimizations.

## Background

Hemocytes are the primary immune cells in insects, including *Anopheles gambiae* mosquitoes, the major vector of the malaria-causing *Plasmodium* parasite [1, 2]. The study of hemocytes has recently gained importance for understanding the mechanistic details of the mosquito innate immune system and its role in eliminating pathogens to limit disease transmission [3–6]. Based on their morphology, mosquito hemocytes have been classified into three distinct cell types: precursor prohemocytes, phagocytic granulocytes, and oenocytoids, which mediate melanization responses [7]. Single-cell RNA sequencing (scRNA-seq) has emerged as a powerful and highly sensitive tool to characterize cells based on unique gene expression profiles and has gained tremendous momentum in uncovering the functional heterogeneity and novel subpopulations of mosquito hemocytes [8–10]. In addition, bulk mRNA sequencing is now an indispensable tool for investigating global transcriptomic responses in hemocytes to different pathogens and to specific physiological conditions [11–13].

Performing both single-cell and bulk transcriptomic analyses of hemocytes from adult mosquitoes is quite challenging because these cells are small, have low RNA content, are highly reactive, and are present in limited numbers in the mosquito hemolymph. Furthermore, circulating hemocytes are obtained by hemocoel perfusion of individual mosquitoes with a buffer. This procedure causes damage to the thoracic region that can lead to contamination with other cells from loosely organized, fragile tissue, such as fat body trophocytes [14], or with ambient RNA released into the hemolymph by damaged tissues. Therefore, major challenges associated with hemocyte RNA-seq include alterations in gene expression and the risk of RNA degradation during the extended period of sample collection, as well as contamination with RNA from other cells present in the perfused sample. These issues can result in undesired data variability and loss of fidelity, as well as the introduction of technical artifacts that severely impact data quality and can complicate the interpretation of the results. Thus, it is essential to optimize a robust protocol for scRNA-seq and bulk RNA-seq of hemocytes to minimize potential technical artifacts and generate high-quality, reproducible data.

Here, we developed a protocol for single-cell and bulk RNA sequencing analysis from stabilized hemocytes of adult female *Anopheles gambiae* mosquitoes. Hemocytes are stabilized for single-cell RNA sequencing by immediately placing the perfused solution in vivoPHIX^®^, a deep eutectic solvent, and, for bulk sequencing, RNA degradation is prevented with the RNAprotect Cell Reagent^®^. These treatments stabilize cellular components and preserve the transcriptomic landscape of hemocytes at the time of sampling and make it feasible to store samples and process multiple groups of samples together, thereby minimizing batch effects and technical variability. The perfusion method of hemocyte collection used in this protocol yields a higher number of cells (5000–10000 hemocytes per sugar-fed mosquito) compared to other reports and techniques (500–4000 hemocytes per sugar-fed mosquito) [7, 15].

The stabilization of hemocytes for single-cell RNA sequencing using vivoPHIX, followed by the ethanol-based recovery protocol developed in this study, is compatible with downstream multi-omic applications including scRNA-seq library preparation using the 10X Genomics platform. It results in the recovery of a higher number of cells compared to other reported protocols, such as using SMART-seq2 single-cell technology with live hemocytes that require immediate sample processing [8–10]. SMART-seq2 is a plate-based single-cell method that typically isolates live hemocytes by FACS, whereas 10X Genomics captures individual cells in nanoliter-scale GEMs using microfluidics. Live hemocyte sorting also requires staining with dyes, which may introduce undesirable transcriptional changes, affect cell viability, and thereby compromise data quality and cell recovery. The strength of scRNA-seq data relies on the number and quality of cells included in the analysis. Clustering analysis performed with a larger number of high-quality cells is crucial for identifying rare cell populations and detecting transcriptomic differences between clusters with higher confidence. Once samples are stabilized for bulk RNA-seq, it is possible to enrich for hemocytes by removing larger cells or tissue fragments present in the perfused samples through filtration with different size cutoffs. This is followed by hemocyte recovery via centrifugation, which reduces contamination with ambient RNA and enriches for hemocyte-specific transcripts during downstream library preparation, thereby improving sample quality and facilitating data acquisition and analysis.

Furthermore, stabilized hemocytes can be stored at 4 °C for up to 4 weeks in both preservative solutions, facilitating easy sample storage and transportation. It is to be noted that the recovery of small hemocytes such as prohemocytes (< 5 μm in diameter) is lower (20–30%) as compared to larger hemocytes such as oenocytoids and granulocytes, which could introduce a potential cell-type bias during sample preparation for single-cell RNA sequencing. In addition, it is also necessary to pool hemocytes from several mosquitoes to obtain enough hemocytes for single-cell library preparation and minimize the variation of hemocyte composition among individual mosquitoes. Nevertheless, the protocol was validated for single-cell transcriptome analysis of adult female *An. gambiae* mosquito hemocytes, and it led to the identification of several prohemocyte and granulocyte subpopulations based on the expression of unique molecular markers [16]. In the same study, the protocol for bulk transcriptome analysis of stabilized samples was also successfully used for in-depth global transcriptomic analysis of hemocyte responses to modulation of specific immune signaling pathways [16]. Altogether, the protocols for single-cell and bulk transcriptomic analysis of stabilized hemocytes will enable a deeper understanding of mosquito immune responses, driving discoveries in vector biology and disease control.

## Materials

### Biological materials

- Adult female*Anopheles gambiae*mosquitoes: The*Anopheles gambiae G3*strain used in this study was obtained from BEI Resources (catalog no. MRA-112). Mosquitoes were reared at 27 °C and 80% humidity under a 12-hour light/dark cycle. 4- 6-day-old female mosquitoes were used for the study.
- Mouse strain: Female BALB/c mice were used in this study. BALB/c mice were purchased from Charles River Laboratories, and mice aged 4–6 weeks were used for mosquito feeding.
  Mouse studies were performed in an Association for Assessment and Accreditation of Laboratory Animal Care (AAALAC)-accredited facility at the National Institutes of Health according to the Institutional Animal Care and Use Committee (IACUC)-approved protocol (LMVR-5). In brief, mice are group-housed in Allentown NexGen IVC cages of 5 mice or less per cage. Health and environmental monitoring are performed twice daily, and cage changing is performed weekly. Mice are provided with water and feed (LabDiet^®^5V0TAdvanced Protocol Verified – 75 IF/Auto) ad libitum. Aspen chew blocks (Bioserv) and nestlets (Ancare) are provided at all times for enrichment. The animal holding room is maintained at 72 degrees F +/- 3, with 12 h light/12 hours dark cycles, and humidity is maintained at 30–70%.

### Materials required for blood-feeding mosquitoes on mice

#### Reagents

1. 1 ml syringe with 25-gauge needle (Thermo Fisher Scientific, Catalog14-817-125).
2. Xylazine hydrochloride (100 mg/mL, Rompun^®^, Dechra Veterinary products, NDC 17033-099-05).
3. Ketamine hydrochloride (100 mg/mL, Zetamine™, VetOne, NDC 13985-584-10).
4. 5 M NaCl (KD Medical, Catalog RGF-3270).
5. Puralube^®^ ophthalmic ointment (Dechra Veterinary Products, NDC 17033-211-38).
6. LabDiet^®^ 5V0TAdvanced Protocol Verified – 75 IF/Auto (LabDiet, Catalog 3004624-703).
7. Aspen chew blocks (Bioserv, Catalog-K3640).
8. Nestlets (Ancare, Catalog- NES3600).
9. NexGen IVC cages (Allentown, Catalog SKU 232822-1).

#### Composition of anesthetic reagent: 1.2 mL

**Table:** 

Reagent	Concentration	Amount
Xylazine hydrochloride	100 mg/mL	40 µL
Ketamine hydrochloride	100 mg/mL	400 µL
NaCl	0.9%	760 µL

- Store the anesthetic reagent at room temperature for up to 1 week.

### Materials required for collecting hemolymph from mosquitoes

#### Reagents

1. Schneider *Drosophila* media (Lonza, catalog 04-351Q).
2. 1 N NaOH (Thermo Scientific, Catalog 124260010).
3. 0.5 M EDTA (Thermo Scientific, Catalog AM9260G).
4. 5 M NaCl (KD Medical, Catalog RGF-3270).
5. Citric acid (Millipore Sigma, Catalog 251275).
6. Molecular biology grade water (Thermo Scientific, Catalog 10977015).
7. Mineral oil (Sigma, Catalog M8410-500mL).
8. Sigmacote to prepare siliconized tips (Sigma Aldrich, Catalog SL2).

#### Laboratory supplies

1. 1.7 mL microcentrifuge tubes.
2. Ice bucket and ice.
3. Glass Petri dishes 100 mm (VWR, Catalog 25354-069).
4. A pair of Fine dissection forceps (Dumont #5 Forceps, FST, Catalog 11252-30).
5. Kimwipes.
6. Thin-walled single-barrel borosilicate glass capillaries (World Precision Instruments, Catalog TW150-4).
7. Flexible tubing (McMaster-Carr, Catalog 1883T4).
8. 10µL Siliconized pipette tips.
9. 1000µL tips.
10. Wipes with 70% Ethanol.
11. Syringe filter 0.22 μm (Fisher Scientific, Catalog 09-720-3).
12. 50 ml Syringe for filling the capillary needle (BD Precision Glide, Catalog 305165).

#### Laboratory equipment

1. Dissection microscope.
2. pH meter.
3. Narishige PC-100 Vertical Needle puller.
4. Volumetric pipettes.
5. Micromanipulator support base, micromanipulator (Drummond Scientific Company, Catalog 3-000-025-SB, 3-000-024-R).
6. Hamilton PB600-1Repeating dispenser (Hamilton, catalog Part # 83700).
7. Needle holder (Tritech Research, Catalog UP-1).

#### Solution recipes

##### Citrate buffer (100mL)

98mM NaOH, 186mM NaCl, 1.7mM EDTA, 41mM citric acid, pH 4.5. Prepare the buffer with sterile molecular biology-grade water and adjust the pH to 4.5. Sterilize the buffer by filtering through a 0.22 μm filter.

- Store the citrate buffer at 4 °C for up to 12 months.

**Table:** 

Reagent	Amount	Final concentration
Citric acid	7.876 g	41mM
1 N NaOH	9.8 mL	98mM
5 M NaCl	3.72mL	186mM
0.5 M EDTA	340µL	1.7mM
Molecular biology grade water	Up to 100 mL	

##### Hemolymph collection buffer (100mL)

95% Schneider Drosophila media (Lonza, catalog 04-351Q) and 5% citrate buffer, pH 7.0-7.4.

Mix Schneider’s media with citrate buffer and set the pH of the solution to 7. Filter the buffer with a 0.22 μm filter to sterilize it.

- Store the hemolymph collection buffer in aliquots at 4 °C for up to 6 months.

**Table:** 

Reagent	Volume	Final concentration
Schneider Drosophila media	95 mL	95%
Citrate buffer	5 mL	5%

### Materials required for performing scRNA-seq post-hemolymph collection

#### Reagents and kits

1. vivoPHIX (Rapid Labs, RNAssist Ltd, Cambridge, UK, Catalog RD-VIVO-50).
2. Ethyl alcohol (Sigma Aldrich, Catalog- E7023-1 L).
3. Nuclease-free water (not DEPC-treated) (Thermo Fisher Scientific, catalog AM9937).
4. Ultrapure BSA (Thermo Scientific, Catalog- AM2618).
5. Chromium Next GEM Single cell 3’ GEM, Library and Gel Bead kit v3.1,16 reactions (10X Genomics, Catalog- PN-1000268).
6. Dual Index Kit TT Set A, 96 reactions (10X Genomics, Catalog- PN-1000215).
7. High Sensitivity D5000 ScreenTape (Agilent, Catalog- 5067–5592).
8. High Sensitivity D5000 Ladder and Sample buffer (Agilent, Catalog-5067-5593).
9. SPRIselect beads (Beckman Coulter Inc, Catalog- B23318).

#### Laboratory supplies

1. Pluriselect mini cell strainer-70 μm (pluriselect, Catalog- 43-10070-50).
2. Pluriselect mini cell strainer-40 μm (pluriselect, Catalog- 43-10040-50).
3. 1.7 ml microcentrifuge tubes.
4. 1000µL tips.
5. 200µL tips.
6. 20µL tips.
7. 2µL tips.
8. Optical Tube 8X strip (Agilent, Catalog 401428).
9. Optical Cap 8X Strip (Agilent, Catalog 401425).
10. Adaptors for holding 8X strip tubes.
11. Neubauer improved disposable hemocytometer (Fisher Scientific, Catalog- 22-600-100).

#### Laboratory equipment

1. Refrigerated centrifuge (Eppendorf, Model- 5425R).
2. TapeStation system.
3. Vortex mixer.
4. Heating bath.
5. Bench-top centrifuge.
6. Chromium controller.
7. Illumina NovaSeq 6000 sequencing instrument.
8. qPCR instrument.
9. Microscope.

### Materials required for isolating RNA for bulk sequencing post-hemolymph collection

#### Reagents and kits

1. RNAprotect Cell Reagent (Qiagen, Catalog-76526).
2. Trizol LS reagent (Invitrogen, Catalog- 10296028).
3. Chloroform (Sigma Aldrich, Catalog- 288306).
4. Isopropanol/ 2-Propanol (Sigma Aldrich, Catalog- I9516-1 L).
5. Linear acrylamide (Thermo Fisher Scientific, Catalog- AM9520).
6. Ethyl alcohol (Sigma Aldrich, Catalog- E7023-1 L).
7. Nuclease-free water (not DEPC-treated) (Thermo Fisher Scientific, catalog AM9937).
8. High Sensitivity RNA ScreenTape (Agilent, Catalog- 5067–5579).
9. High Sensitivity RNA ScreenTape Ladder (Agilent, Catalog-5067-5581).
10. High Sensitivity RNA ScreenTape Sample buffer (Agilent, Catalog- 5067–5580).
11. 7-AAD dye (Invitrogen, Catalog A1310, Working concentration 1 µg/mL).
12. DyNAmo HS SYBR Green qPCR Kit (Thermo Fisher Scientific, catalog F410XL).
13. DAPI (Molecular Probes, Catalog D1306).
14. Primers LRR8 Forward: TCTGTCGAGCTGCGTAGTTG, Reverse: AGGTCCTCCAGCAGTGGTAA.
15. Primers S7 Forward: AGAACCAGCAGACCACCATC, Reverse: GCTGCAAACTTCGGCTATTC.
16. NEBNext^®^ Poly(A) mRNA Magnetic Isolation Module (New England Biolabs, Catalog- NEB# E7490).
17. NEBNext Ultra II RNA Non-directional Library Prep Kit for Illumina (New England Biolabs, Catalog- NEB# E7770).

#### Laboratory supplies

1. Pluriselect mini cell strainer-70 μm (pluriselect, Catalog- 43-10070-50).
2. Pluriselect mini cell strainer-40 μm (pluriselect, Catalog- 43-10040-50).
3. Phasemaker™ tubes (Invitrogen, Catalog- A33248).
4. 1.7 ml microcentrifuge tubes.
5. 1000µL tips.
6. 200µL tips.
7. 20µL tips.
8. 2µL tips.
9. Optical Tube 8X strip (Agilent, Catalog 401428).
10. Optical Cap 8X Strip (Agilent, Catalog 401425).
11. Adaptors for holding 8X strip tubes.

#### Laboratory equipment

1. Refrigerated centrifuge.
2. TapeStation system.
3. Vortex mixer.
4. Heating bath.
5. Quantitative PCR detection system.
6. Illumina NovaSeq 6000 sequencing instrument.

#### Software and resources for data analysis

1. CellRanger v7.0.0.
2. decontX.
3. Seurat v4.3.0.
4. FastQC v0.12.0.
5. TrimGalore v0.6.7.
6. RSEM v1.3.3.
7. Bowtie 2.
8. R.
9. edgeR.
10. Scripts: Zenodo repository at https://doi.org/10.5281/zenodo.18224827.
11. Hemocyte transcriptome annotation is available at https://proj-bip-prodpublicread.s3.amazonaws.com/transcriptome/An_gambiae_hemocytes_2022/AgHemocytes.zip.

## Procedure

### Prepare before beginning the experiment

1. Siliconize micropipette tips and 1.5 mL centrifuge tubes at least a day before hemolymph collection.
2. Prepare glass capillary needles for perfusing the hemolymph.
3. Prepare the pH-adjusted and sterile-filtered hemolymph collection buffer.
4. Set the hemolymph perfusion system under the dissection microscope at least 15 min before starting the hemolymph collection from the mosquitoes to stabilize the system.
5. Aliquot the required volume of the hemolymph collection buffer and place it on ice before beginning the perfusion.
6. Place a glass Petri dish on ice to cool for anesthetizing mosquitoes prior to hemolymph collection.
7. Pre-cool the centrifuge to 4 °C before starting RNA isolation from stabilized hemocytes.

#### Blood-feeding mosquitoes on mice

**Time: 30–40 min**

1. Place the mosquitoes to be fed in a separate confined cage with net on top, with 60–80 mosquitoes in each cage.
2. Starve the mosquitoes for 12–16 h by replacing sugar meal with a cotton ball dipped in sterile distilled water.
3. Remove the cotton ball at least 4 h before the blood feeding.
4. Anesthetize the mouse by administering the anesthetic reagent intra-muscular at the leg at a final concentration of Xylazine- 6.66 mg/kg and Ketamine 66.6 mg/kg of body weight of the mouse (approximately 50 µL for a mouse with 25-gm body weight).
5. After the mouse is completely sedated, place the mouse on the mosquito cage with their abdomen facing the mosquitoes for up to 30 min. Apply Puralube on the mouse eyes to prevent desiccation.
6. Perform the feeding at room temperature and monitor the mouse during feeding.
7. At the end of feeding, euthanize the mice by administering a higher dose of anesthetic reagent (100 mg/kg Ketamine and 10 mg/kg Xylazine) to deeply sedate the mouse and cervical dislocation under sedation.

#### Siliconizing centrifuge tubes and micropipette tips

**Time: 15 min**

**Note**: Sigmacote is a toxic reagent and should be used inside a chemical fume hood, and the plasticware should be dried completely post-coating before use.

1. Aliquot 1mL of Sigmacote in a 1.7 ml centrifuge tube.
2. For coating 1.7 ml centrifuge tubes, arrange the tubes in a stand with their lids open and add 1mL of the aliquoted Sigmacote in each tube with the help of a micropipette and transfer all the liquid into the next tube immediately.
3. Repeat the step for all the tubes to be coated. At the end of the process, leave the tubes on the rack with the lid kept open overnight to allow the tubes to dry.
4. For preparing coated micropipette tips, pipette up and down two times the Sigmacote into each tip, and then transfer the tip into a rack and allow them to dry overnight before use.

- Store the coated tips and tubes at room temperature in sealed containers for up to 8 months.

#### Setting up the hemolymph perfusion system

**Time: 20 min**

**Fig. 1:**
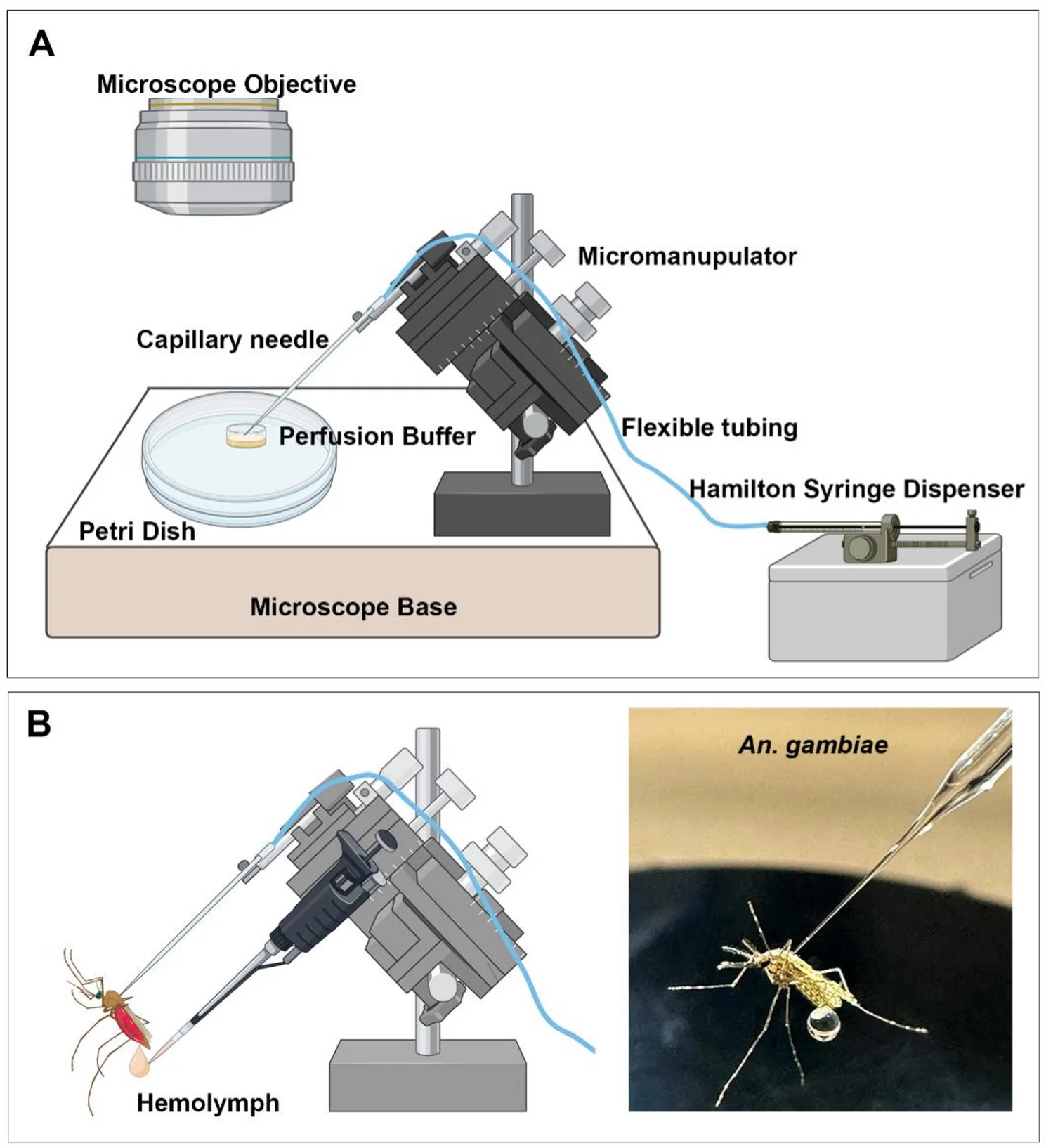
Schematic of the hemolymph perfusion system. (**a**) Pictorial representation of the lay-out of multiple components to perfuse hemolymph from mosquitoes. (**b**) (Left) Diagram showing the perfused hemolymph forming a drop at the end of the mosquito abdomen, (Right) actual image of hemolymph perfused from a female *An. gambiae* mosquito

1. Prepare glass capillary needles using Narishige needle PC-100 puller using thin-walled single-barrel borosilicate glass capillaries (World Precision Instruments, Catalog TW150-4). Use the puller instrument under the following setting conditions: Heating level 1 set at 68 °C, and press the “Step 1” button. Break open the end of the glass capillary needle with fine forceps, keeping the needle length in the tapered region of approximately 6–8 mm. The diameter of the obtained needle is approximately 0.3–0.4 mm.
2. Connect the micromanipulator to the support base and attach the needle holder to the micromanipulator. Place the setup on the base of a dissection microscope.
3. Take one capillary needle and connect the flexible tubing (approximately 30–50 cm long) to the base of the needle. Then attach the needle to the micromanipulator at approximately a 45–60° angle with the help of the needle holder.
4. With the help of a syringe needle, fill the capillary needle and the tubing with mineral oil, and connect the tube to a Hamilton PB-600 Repeating Dispenser filled with mineral oil.
5. Adjust the setup to perfuse the mosquitoes for hemolymph collection under a dissection light microscope (Fig. 1A).

#### Hemolymph collection from the mosquitoes by perfusion

**Time: 2–3 min per mosquito**

1. Place a small sterile dish on the lid of a closed Glass Petri dish filled with ice. Add a small volume (400–500µL) of the hemolymph collection buffer to the sterile dish.
2. Draw ice-cold hemolymph collection buffer into the capillary needle with the help of the Hamilton dispenser.
3. Place an anesthetized mosquito on the glass Petri dish by lying it down on the side.
4. Clean the forceps tips with alcohol wipes and make a small incision near the last abdominal section of the mosquito by gently separating the segments.
5. Insert the capillary needle tip intrathoracically above the thorax-abdomen junction of the mosquito by maneuvering the micromanipulator, and lift the mosquito attached to the needle slightly above the Petri dish surface.
6. Gently pass 5–6 µL of buffer through the hemocoel of the mosquito with the Hamilton Repeating dispenser by pressing 2–3 times. The diluted hemolymph forms a drop at the abdominal incision (Fig. 1B).
7. Collect the hemolymph with siliconized tips and add it directly to a 1.7mL tube containing the appropriate stabilizing solution.

#### Procedure for hemolymph collection and stabilization for scRNA-seq library preparation

**Time: Hemolymph collection- 2–3 min per mosquito**,

**Stabilization- 1–1.5 h post collecting hemolymph from the last mosquito**

**Fig. 2:**
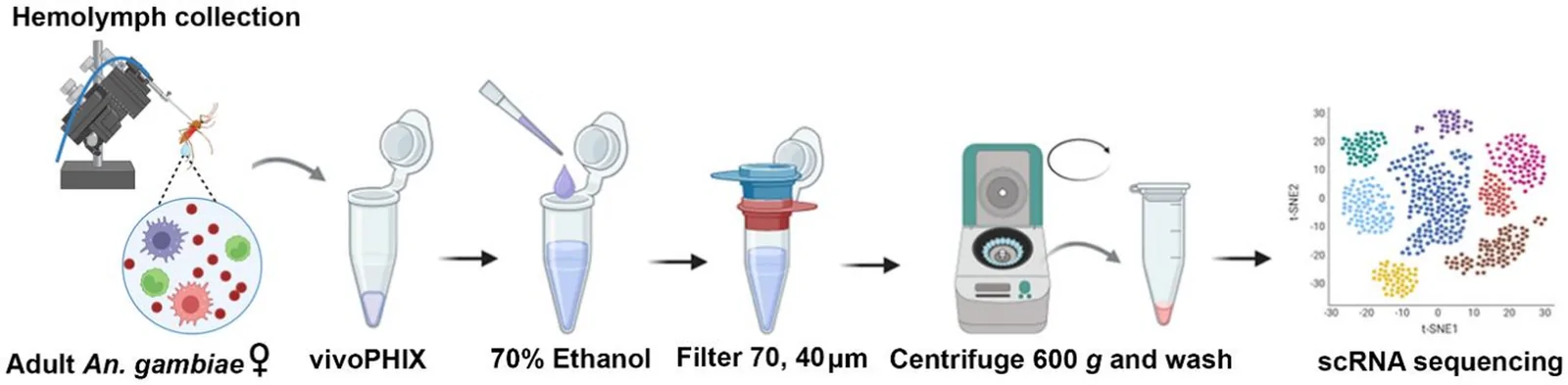
Single-cell RNA sequencing of stabilized hemocytes from adult female *An. gambiae* mosquitoes

The main steps for single-cell RNA sequencing of hemocytes are provided in a schematic (Fig. 2).

1. Collect the hemolymph as described earlier with siliconized tips and add directly to a 1.7 mL tube containing 250 µL of vivoPHIX kept at room temperature.
2. Collect a total of 10 µL of hemolymph from each mosquito and hemolymph of 10 mosquitoes (approx. 100 µL) per 250 µL of vivoPHIX.
3. Mix the hemolymph and vivoPHIX in the tube after bleeding every 3–4 mosquitoes by inverting the tubes.
4. After collecting hemolymph from 10 mosquitoes, mix the solution by inverting the tube and incubate at room temperature for 1 h while mixing 2–3 times at regular intervals to stabilize the hemocytes in the vivoPHIX reagent. The final volume is approximately 350–400 µL.
5. Transfer the samples to 4 °C post-incubation at room temperature until further use.

#### Procedure to recover hemocytes post vivoPHIX stabilization

**Time: 1–1.5 h**

1. Freshly prepare 75% ethanol solution with nuclease-free water and add 500 µL of 75% ethanol solution to the tube containing hemocytes stabilized in vivoPHIX.
2. Mix the solution by pipetting up and down with a 1000 µL tip so that the vivoPHIX containing the stabilized hemocytes is completely diluted with the ethanol.
3. Filter the solution using a 70 μm followed by a 40 μm cell strainer, collecting the filtrate in a new 1.5mL centrifuge tube at room temperature.
4. Centrifuge the tube at 650 *g* for 15 min at room temperature. A small white pellet is obtained.
5. Wash the samples with 1mL of 75% ethanol without resuspending the pellet and centrifuge the tube at 650 *g* for 5 min at room temperature to remove any remaining vivoPHIX.
6. Wash the pellet with 1 mL of freshly prepared 0.1% BSA solution with nuclease-free water by resuspending and spinning at 650 *g* for 15 min at room temperature to remove any remaining ethanol and salts.
7. An invisible pellet is obtained at this step, and the operator should rely on the expected pellet position based on centrifuge orientation and geometry to avoid loss of the pellet. Therefore, carefully discard the supernatant without touching the pellet area.
8. Each 1.7 ml tube yields a cell pellet from approximately 10 mosquitoes. Dissolve and pool the cell pellets obtained from 50 to 60 mosquitoes in 0.1% BSA solution with a final volume of 40–50 µL. This results in a cell count ranging from 700 to 1000 cells per µL.
9. Place the cell suspension on ice till loaded on the scRNA-seq library preparation reaction.
10. Take 10 µL of the cell suspension in a Neubauer-improved disposable hemocytometer and count the cells to estimate the concentration. DAPI staining can also be used to distinguish the cells from debris.
11. Proceed with the single-cell RNA sequencing library preparation with 10,000 cells using the Chromium Next GEM Single Cell 3’ Reagent kit v3.1 (10X Genomics) following the manufacturer’s protocol (Version v3.1 Chemistry Dual Index, User guide CG000315_RevF) with the following modification at Step 2.2.d as mentioned below.
12. Prepare dual-indexed sequencing libraries using the Dual Index Kit TT Set A kit (10X Genomics) following the manufacturer’s protocol (Version v3.1 Chemistry Dual Index, User guide CG000315_RevF) with the following modification at Steps 3.6.a-q as mentioned below.
13. Perform pooled sequencing of the libraries on the NovaSeq 6000 instrument, following the manufacturer’s protocol for Illumina sequencing platforms.
14. Set the following run parameters for library sequencing as specified in the Chromium Next GEM Single Cell 3’ Reagent kit protocol (Version v3.1 Chemistry Dual Index, User guide CG000315_RevF): Read1-28 cycles, i7 index-10 cycles, i5 index-10 cycles, Read2-90 cycles. Use 1% PhiX for sequencing control. The read length is 90 bases and target a sequencing depth of 150 million reads per library.

##### Bioinformatic analysis workflow of single-cell RNAseq data

1. Align sequencing reads to the *Anopheles gambiae* PEST P4.14 reference genome using CellRanger v7.0.0.
2. Generate gene counts using gene models derived from the custom hemocyte transcriptome annotation [18] and create a custom genome reference using the ‘cellranger mkref’ command with the P4.14 FASTA file and a hemocyte-specific GTF annotation file.
3. Perform expression quantification using ‘cellranger count’ with the parameters --include-introns=false and --expectcells = 10,000.
4. Since 10,000 cells were used to generate the libraries, define the top 20,000 GEM wells ranked by total UMI count as cells, and treat the remaining wells as empty droplets to estimate background RNA.
5. Analyze the level of ambient RNA contamination using the decontX tool available at (https://doi.org/10.1186/s13059-020-1950-6) and discard cells with decontX contamination estimate values higher than 0.5.
6. Perform downstream analysis using Seurat v4.3.0. Apply the following filters: mitochondrial gene expression < 15%, detected genes 250–2500 (except *dsCactus*, 250–4000 genes) per cell for each sample.
7. Log-normalize each sample using the ‘NormalizeData’ function and identify the top 2,000 most variable genes using the ‘FindVariableFeatures’ function.
8. Determine integration anchors using the Canonical Correlation Analysis reduction method with the ‘FindIntegrationAnchors’ function.
9. Integrate the cells from different samples using the ‘IntegrateData’ function to obtain a combined dataset.
10. Scale the combined dataset using the ‘ScaleData’ function and perform dimensionality reduction with ‘RunPCA’ function, retaining the top 30 principal components.
11. Compute a UMAP embedding using ‘RunUMAP’ based on the top 30 PCs and perform clustering using ‘FindClusters’ with a resolution of 0.1.
12. Remove clusters corresponding to fat body cells and analyze the remaining hemocyte cells using the same normalization, integration, and clustering workflow.
13. Perform Final clustering using ‘FindClusters’ with its ‘resolution’ parameter set to 0.3.
14. Identify Cluster marker genes using FindAllMarkers with parameters set to min.pct = 0.25, logfc.threshold = 0.5, and test.use = ‘wilcox’.
  All the scripts used in the data analysis are deposited in the Zenodo repository at https://doi.org/10.5281/zenodo.18224827, and the analysis workflow is published in the referenced article [16].

#### Procedure for hemolymph collection and processing for bulk RNA sequencing

**Time: Hemolymph collection- 2–3 min per mosquito**,

**RNA isolation- 2–3 h**,

**RNA quality check- 30 min**

**Fig. 3:**
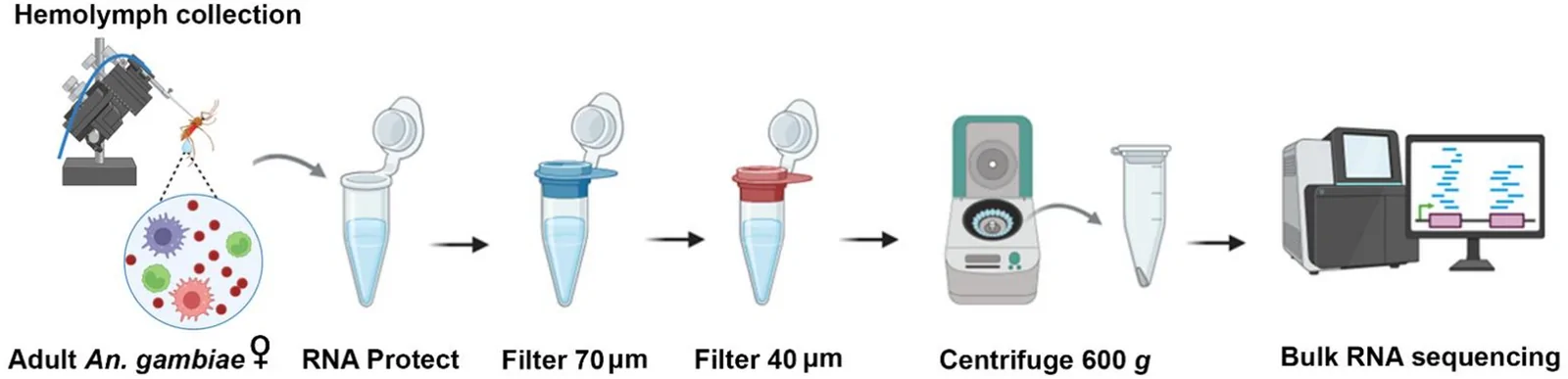
Bulk RNA sequencing of stabilized hemocytes from adult female *An. gambiae* mosquitoes

The main steps for isolating high-quality RNA from hemocytes bulk RNA sequencing are provided in a schematic (Fig. 3).

1. Collect the hemolymph with siliconized tips as described above and add directly to a 1.7mL tube containing 800 µL of RNAprotect Cell Reagent kept at room temperature.
2. Collect a total of 8 µL of hemolymph from each mosquito and hemolymph of 20 mosquitoes (approx. 160 µL) per 800 µL of RNAprotect Cell Reagent.
3. Mix the hemolymph with RNAprotect Cell Reagent after bleeding every 3–4 mosquitoes by gently inverting the tubes and avoiding bubble formation.
4. After collecting hemolymph from the last mosquito, incubate the tube at room temperature for 1 h to stabilize the hemocytes in the RNAprotect Cell Reagent.
5. Transfer the samples to 4 °C until further use post-incubation at room temperature.
6. If proceeding for cell lysis, filter the solution through a 70 μm followed by a 40 μm cell strainer.
7. Collect the filtrate in a new 1.5 mL tube and centrifuge the tubes at 4 °C, at 650 *g* for 15 min. A small white pellet is observed.
8. Discard the supernatant without disturbing the pellet and leaving 20–30 µL of liquid at the bottom of the tube.
9. Dissolve the pellet in the remaining liquid by gently tapping on the sides of the tube. Then add 1 mL of Trizol LS reagent to the tube and mix well to ensure cell lysis.
10. Store the samples at -80 °C or proceed with RNA isolation following the next steps.
11. Take Phasemaker™ tubes, one per sample, and spin them at 11,000 g for 5 min.
12. Add 200 µL of Chloroform to the tube containing hemocyte lysate in Trizol and mix by inverting the tube 15–20 times.
13. Transfer the solution to the prespun Phasemaker™ tube with help of a micropipette and centrifuge at 12,000 *g* for 15 min at 4 °C to separate the aqueous and organic phases.
14. Collect the transparent aqueous phase formed above the gel in a new tube and add linear acrylamide at a final concentration of 20 µg/mL.
15. Mix the linear acrylamide well by pipetting and then add 500 µL of Isopropanol.
16. Mix the solution by inverting the tubes and incubate at room temperature for 40–45 min followed by centrifugation at 12,000 *g* for 15 min at 4 °C.
17. A small pellet of the RNA is observed. Discard the supernatant carefully and add 1 mL of ice-cold 70% ethanol to the pellet to wash the RNA.
18. Centrifuge at 12,000 *g* for 15 min at 4 °C and discard the supernatant. Repeat the wash step one more time.
19. After the second wash, carefully discard all the liquid and allow the pellet to dry. The pellet turns transparent upon drying.
20. Add 15–20 µL of nuclease-free water warmed at 65 °C to the pellet as soon as the pellet becomes dry and resuspend the pellet by pipetting.
21. Check the quality and concentration of RNA with the help of a Bioanalyzer or Agilent TapeStation instrument, following the manufacturer’s protocol.
22. Store the RNA samples at -80 °C until proceeding with bulk RNA sequencing library preparation. The RNA samples can be stored up to 4 months at -80 °C.
23. Prepare bulk RNA sequencing library using NEBNext^®^ Poly(A) mRNA Magnetic Isolation Module (protocol Version 11.0_2/24) and NEBNext Ultra II RNA library preparation kit (protocol Version 6.0_4/25) to generate non-directional poly (A)- enriched RNA libraries following the manufacturer’s protocols without any alterations.
24. Perform paired-end sequencing of the pooled libraries on an Illumina NovaSeq 6000 platform according to the manufacturer’s protocol without any alterations. Target a sequencing depth of 50 million reads per library using 150-bp paired-end reads.

##### Bioinformatic analysis workflow of bulk RNAseq data

1. Determine the quality of raw Illumina reads using the FastQC tool (version 0.12.0) available at the website https://www.bioinformatics.babraham.ac.uk/projects/fastqc/.
2. Remove low-quality sequences with a Phred quality score (Q) below 20 and Illumina adaptors using the TrimGalore software (v.0.6.7) available at the website https://github.com/FelixKrueger/TrimGalore.
3. Align the high-quality reads to the custom hemocyte-specific transcriptome annotation using RSEM v.1.3.3 with Bowtie 2 as the aligner for transcript annotation. The hemocyte-specific transcriptome annotation is available at https://proj-bip-prodpublicread.s3.amazonaws.com/transcriptome/An_gambiae_hemocytes_2022/AgHemocytes.zip.
4. Use raw read counts for each transcript as input for the edgeR package for differential expression analysis.
5. Normalize the counts using the trimmed mean of M-values (TMM) method to account for compositional differences between libraries.
6. Perform a quasi-likelihood F-test using the glmQLFTest function, following model fitting with glmQLFit in edgeR, to account for gene-specific variability in dispersion estimates and to provide more reliable control of the false discovery rate. For this bulk dataset, transcripts were considered differentially expressed if they exhibited an absolute Log2FoldChange: |log2FC| ≥ 1 and a false discovery rate (FDR) < 0.05.
  All the scripts used in the data analysis are deposited in the Zenodo repository at https://doi.org/10.5281/zenodo.18224827 and the analysis workflow is published in the referenced article [16].

## Troubleshooting

**Table:** 

Issue	Remedy
**A. Setting up the hemolymph perfusion system**	
Perfusion pressure is low due to multiple air bubbles trapped in the tube and < 3 µL of diluted hemolymph is obtained at step 4.6.	Disconnect the tube from the Hamilton syringe dispenser and remove the capillary needle. Reattach a new capillary needle and refill the tube with oil.
Perfusion pressure is too high, and the mosquito drops down from the capillary needle at step 4.6.	Introduce one or two small air bubbles in the tube and press the Hamilton dispenser gently, allowing a small volume of buffer to enter the mosquito hemocoel.
**B. Recovery of hemocytes post vivoPHIX stabilization**	
The pellet obtained at step 6.7 is white.	This occurs due to the presence of salt or debris. Repeat the wash with 1 ml of 0.1% BSA in nuclease-free water.
The cell suspension has excessive debris at step 6.8.	This occurs if the washing of the cell pellet is not optimal or if cells are breaking due to vigorous pipetting pressure. To overcome this, introduce an additional wash step with 1 ml of 0.1% BSA in nuclease-free water and resuspend the cell pellet by applying medium pressure during pipetting.
The concentration of the hemocytes is less than the range recommended by 10X Genomics at step 6.10.	Add 200 µL of 0.1%BSA in nuclease-free water to the cell suspension and centrifuge the cells to obtain a cell pellet. Dissolve the cell pellet in a smaller volume of 0.1%BSA in nuclease-free water.
**C. RNA isolation for bulk RNA sequencing**	
The gel layer in the Phasemaker tube™ is above the aqueous and organic layers at step 7.14.	This is observed due to a low amount of RNA in the aqueous layer. To recover the aqueous layer, gently pierce the gel layer with a pipette tip and carefully collect the aqueous layer without touching the pink organic layer.
Low RNA yield at step 7.21.	To overcome this, collect the hemolymph from a larger number of mosquitoes in the appropriate volume of RNAprotect Cell Reagent for RNA isolation and precipitate the RNA in Isopropanol overnight at -20 °C.

## Expected results

### Single-cell RNA sequencing of hemocytes stabilized with vivoPHIX

**Fig. 4:**
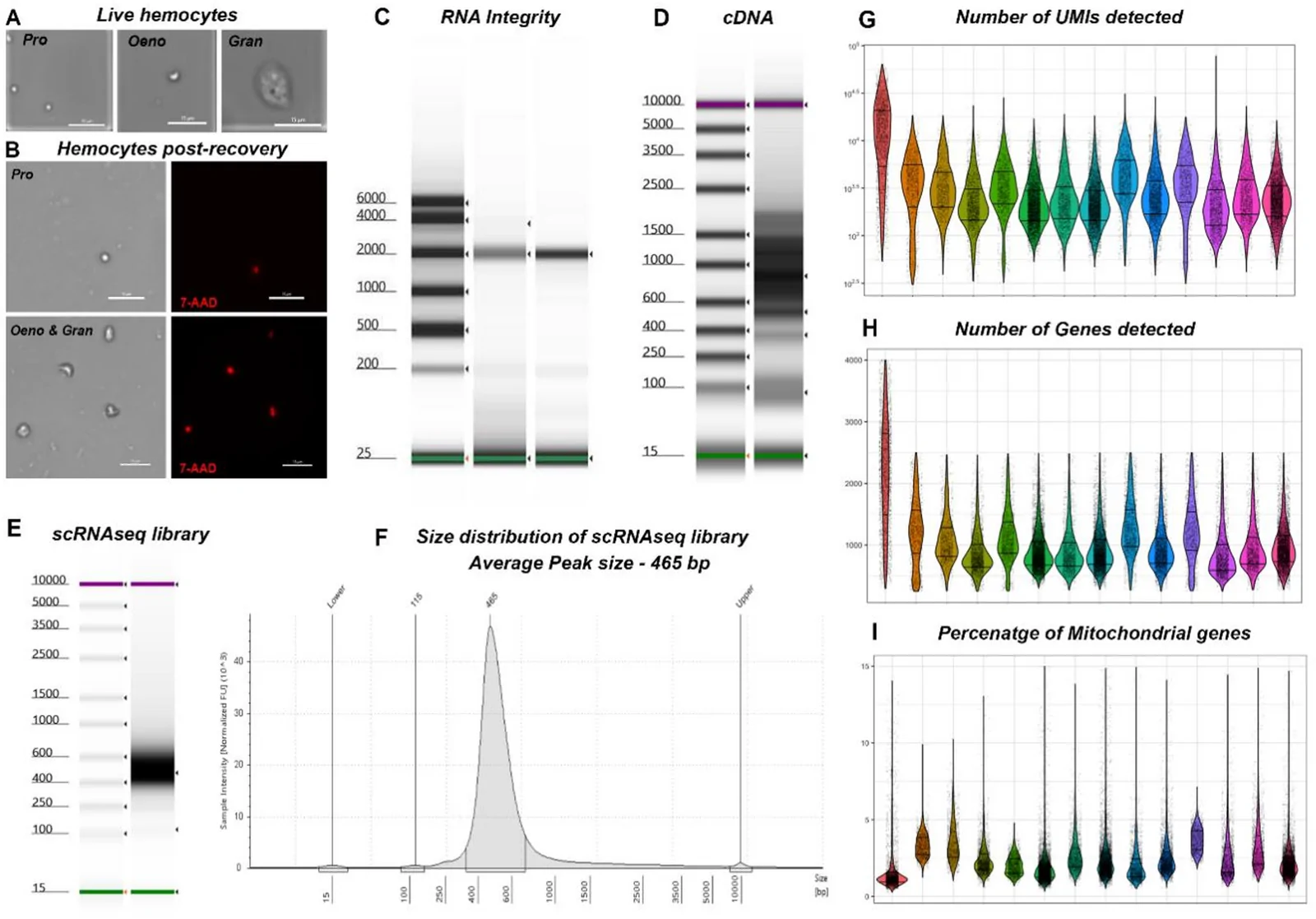
Quality metrics of single-cell RNA sequencing libraries generated from *An. gambiae* hemocytes stabilized with vivoPHIX. (**a**) Bright-field light microscopy images of perfused live hemocytes from adult *An. gambiae* females. (**b**) (Left) Bright-field light microscopy images of hemocytes post vivoPHIX stabilization and recovery, (Right) Fluorescent microscopy images showing hemocyte nuclei stained with 7-AAD. Scale bar- 15 μm. (**c**) Electrophoresis of the RNA isolated from stabilized hemocytes post-recovery performed on Tapestation instrument. (**d**) Electrophoresis of the amplified cDNA during scRNA-seq library preparation of stabilized hemocytes performed on Tapestation instrument. (**e**) Electrophoresis of the scRNA-seq library prepared from stabilized hemocytes performed on Tapestation instrument. (**f**) Electropherogram depicting the size distribution of the scRNA-seq library fragments. (**g**) Violin plot showing the distribution of number of UMIs detected, (**h**) number of genes detected, and (**i**) percentage of mitochondrial genes detected per cell in each of the 14 samples. Pro- Prohemocytes, Oeno- Oenocytoids, Gran- Granulocytes

Perfused live prohemocytes and oenocytoids from adult *An. gambiae* females are less adherent to glass surface and their diameter is less than 10 μm when observed by bright-field light microscopy (Fig. 4A). In contrast, live granulocytes are highly adherent and spread on glass surfaces attaining a diameter of 15–25 μm (Fig. 4A). The morphology and integrity of the vivoPHIX stabilized hemocytes post-recovery were examined by bright-field light microscopy, and the nucleus was stained with a fluorescent dye 7AAD (7-Amino-Actinomycin D) following manufacturer’s guidelines to distinguish hemocytes from cell debris (Fig. 4B). The morphology of prohemocytes and oenocytoids is not distinctly affected by the stabilization and recovery procedures, but granulocytes are smaller (less than 10 μm) than under live conditions (Fig. 4B, left panel). Post-recovery, granulocytes are round and no longer adhere to glass-surface (Fig. 4B, left panel). However, all hemocytes had an intact cell membrane and nucleus after recovery, indicating that the cellular integrity is not compromised (Fig. 4B, right panel). The integrity of the RNA isolated from stabilized hemocytes post-recovery was also assessed during the optimization of the protocol using the High Sensitivity RNA ScreenTape in the Agilent Tapestation 4200 instrument. It was confirmed that high-quality RNA was obtained without degradation (Fig. 4C).

The single-cell RNA sequencing libraries from stabilized hemocytes were generated using Chromium NEXT GEM Single Cell 3’ Kit v3.1 from 10X Genomics following the manufacturer’s protocol. This kit partitions individual cells into nanoliter-scale Gel Beads in Emulsion (GEM) with the help of a microfluidic platform and specifically detects poly-adenylated mRNA. Ten thousand hemocytes were loaded per sample, and 14 single-cell RNA sequencing libraries were generated. The quality of cDNA and final libraries was evaluated using the Agilent Tapestation 4200 instrument before proceeding to cDNA amplification and sequencing. The cDNA amplification step from stabilized hemocytes was carried out for 13 cycles and the size of cDNA fragments ranged between 400 and 2000 bp (Fig. 4D), with concentration ranging between 30 and 100 ng/µL. Dual-index sequencing libraries were generated and the final library cleanup step using SPRIselect beads was carried out twice to minimize the presence of small-size DNA fragments (potential primer adapters, less than 150 bp) which could hinder library sequencing. High-quality libraries were obtained post-clean up with an average size ranging between 450 and 550 bp and DNA concentrations ranging between 20 and 25 ng/µL (Fig. 4E-F).

The sequencing reads from the single-cell RNAseq data were aligned to the *Anopheles gambiae* PEST P4.14 reference genome using CellRanger v7.0.0 [17] to identify mosquito-specific reads. A custom genome reference was built combining the *Anopheles gambiae* PEST P4.14 FASTA file and a hemocyte-specific GTF annotation file to perform gene expression quantification [18]. The quantification of gene expression was performed using ‘cellranger count’ with the parameters --include-introns=false and --expect-cells = 10,000. To estimate background RNA, the top 20,000 GEM wells ranked by total UMI count were defined as cells, while the remaining wells were treated as empty droplets to estimate background RNA. decontX software [19] was implemented to determine the level of ambient RNA in the cells, and cells with a higher than 0.5 decontX value were removed. This step was essential to accurately distinguish small prohemocytes with limited gene expression from empty GEMs or GEMs containing ambient RNA derived from lysed cells, resulting in the retention of a higher proportion of high-quality cells. The downstream analyses were performed using Seurat v4.3.0 software [20]. The following additional quality filters were used: 250–2500 detected genes per cell and a maximum of 15% mitochondrial gene count for most samples (13 out of 14). Specifically, mitochondrial gene proportion is commonly used as a proxy for cell quality and viability, since increased mitochondrial transcript fraction can reflect cytoplasmic RNA loss or cellular stress/death, leading to disproportionate representation of mitochondrial transcripts. One of the samples was collected from female mosquitoes in which Toll signaling was activated by silencing the suppressor Cactus (ds*Cactus* sample), which is known to result in a dramatic increase in the proportion of giant granulocytes (Megacytes) [12]. For this sample, a higher upper threshold of 4,000 detected genes was used, as previous studies have shown that megacytes possess a substantially higher number of UMI counts and express a greater number of genes per cell, showing this protocol is successful in capturing cells with both low and high RNA content [8, 16].

The summary of the quality control metrics of the single-cell RNA sequencing libraries is provided in Table 1. The average yield of hemocytes per sample was approximately 2,600 cells, which is higher than previous reports [8–10]. The comparison of hemocyte yields is provided in Table 2. In addition, the low percentage of mitochondrial genes (below 3%) indicates high-quality of recovered hemocytes. The distribution of number of unique molecular identifiers (UMIs), number of detected genes and percentage of mitochondrial genes in the final set of hemocytes for each of the 14 samples obtained post-analysis is shown in violin plots (Fig. 4G-I). The first sample in each of the violin plots in Fig. 4G-I corresponds to the ds*Cactus* sample, which has a high number of Megacytes. The clustering analysis and further results obtained from this data are described in detail in a previous study [16].

### RNA extraction of hemocytes for bulk Illumina sequencing stabilized with RNAprotect Cell Reagent

**Fig. 5:**
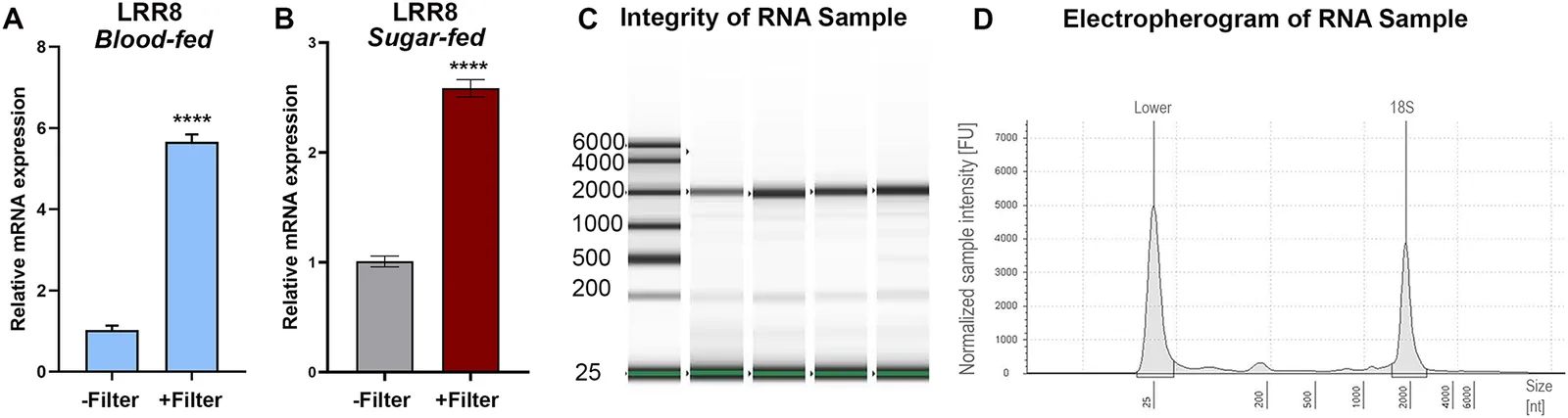
Quality metrics of RNA for bulk sequencing isolated from *An. gambiae* hemocytes stabilized with RNAprotect Cell Reagent. (**a**) Relative mRNA expression of LRR8 in stabilized and filtered hemocytes (+ Filter) compared to total hemolymph (-Filter) isolated from blood-fed mosquitoes. (**b**) Relative mRNA expression of LRR8 in stabilized and filtered hemocytes (+ Filter) compared to total hemolymph (-Filter) isolated from sugar-fed mosquitoes. (**c**) Electrophoresis of the RNA samples isolated from stabilized hemocytes performed on Tapestation instrument. (**d**) Electropherogram indicating the size distribution of an RNA sample isolated from stabilized hemocytes

The quality and integrity of the RNA isolated from the hemocytes stabilized with RNAprotect Cell Reagent and separated from hemolymph were examined. The leucine-rich repeat ficolin (LRR8) encoded by the AGAP004017 gene is expressed at much higher levels in hemocytes than in other tissues from adult *An. gambiae* females [8]. Thus, the normalized expression levels of LRR8 (using ribosomal protein S7 as a reference) in RNA from stabilized hemolymph perfusions can be used to assess the relative abundance of hemocytes in a given sample. The perfused and stabilized hemolymph was filtered through a 70 μm to remove fragments of cuticle, thoracic muscle or trachea, followed by a 40 μm cell strainer to remove cells larger than hemocytes, such as fat body trophocytes, that could be contaminating the sample. The efficiency of these filtering steps to remove contaminating tissues or cells and enrich for hemocyte-specific transcripts was evaluated in samples collected from sugar-fed or blood-fed females 48 h post-feeding (Fig. 5A-B). We found that, indeed, the relative expression of LRR8 was 5.6 times (*p* < 0.0001, Unpaired *t*-test, *n* = 3 biological replicates) higher in the RNA isolated from filtered perfused hemolymph of blood-fed females (Fig. 5A); while in sugar-fed females, expression of LRR8 was 2.5 times higher (*p* < 0.0001, Unpaired *t*-test, *n* = 3 biological replicates) than the unfiltered control (Fig. 5B). This indicates that filtration after sample stabilization reduces contaminating RNA and enriches for hemocyte-specific transcripts.

The integrity of the RNA sample is critical for generating high-quality libraries for bulk Illumina sequencing. The integrity of the RNA isolated from stabilized hemocytes (*n* = 34 samples, sugar-fed mosquitoes) was assessed using the High Sensitivity RNA ScreenTape in the Agilent Tapestation 4200 instrument. Each sample yielded total RNA ranging from 70 to 200 ng with RIN (RNA Integrity Number) values between 8.8 and 9, indicating high-quality RNA without degradation (Fig. 5C). In insects, the 28 S rRNA is composed of two fragments held together by hydrogen bonds and, under denaturing conditions, the two fragments separate and migrate at similar position as the 18 S rRNA [21], thereby exhibiting a single band observed in the gel and the electropherogram (Fig. 5C-D). This is different from the two bands (18 S and 28 S rRNA) typically observed in vertebrate samples.

The quality metrics of the bulk mRNA sequencing libraries generated from the stabilized hemocytes is summarized in Table 3. Post-Illumina sequencing, each library generated 49.72 million total reads with greater than 97% Q20 and 93% Q30 Phred quality scores of base-call accuracy during sequencing, indicating high quality of these libraries. The data obtained from the bulk mRNA sequencing were used to analyze in-depth differential gene expression across different treatments, and the results obtained are reported in the referenced study [16].

The optimized protocol for stabilization and recovery of hemocytes for single-cell RNA seq analysis can be used to perform other multiomic assays such as mRNA and open chromatin region, thereby combining epigenetic and transcriptional regulation in the same assay. It can also be utilized to detect pathogen infection in combination with scRNA-seq analysis to monitor tissue or cell-type tropism. The protocol developed to obtain high -quality hemocyte RNA could also be used for long-read sequencing, which is critical to identify novel isoforms or alternate splice forms of the transcripts.

These protocols will be instrumental for in-depth analysis of several aspects of mosquito hemocytes aiding to design novel vector-borne disease intervention strategies to prevent transmission of mosquito-borne disease. Moreover, this methodology is easily applicable for bulk transcriptomic and single-cell RNA sequencing analysis in other mosquito and insect species.

**Table 1:** Quality metrics of scRNA-seq libraries generated from stabilized hemocytes

Number of scRNA-seq libraries tested	**14**
Cells loaded per sample	**10,000**
Average Cells recovered/ library	**2680 ± 576**
Average Mean reads/ cell	**4053 ± 776**
Average Median genes detected/ cell	**1040 ± 167**
Average % Mitochondrial genes/ cell	**2.57 ± 0.58**

The standard deviation value for each parameter is indicated for each sample after the symbol ±

**Table 2:** Comparison of hemocyte recovery in single-cell RNA sequencing studies

Parameter	Severo et al. 2018 [10]	Kwon et al. 2021 [9]	Raddi et al. 2020 [8]	Saha et al. 2026 [16]
Live/ stabilized hemocytes	Live	Live	vivoPHIX stabilized	vivoPHIX stabilized
Single-cell technology	SMART-seq2	SMART-seq2	10X Genomics Chromium Platform	10X Genomics Chromium Platform
Total cells recovered post-data analysis	56	262	5383	37,529

**Table 3:** Quality metrics of the bulk RNA-seq libraries generated from stabilized *An. gambiae* hemocytes

Number of RNA samples tested	34
Average RNA yield/ sample (in ng)	**95.8 ± 44.8**
Average RNA Integrity Number/sample	**8.8 ± 0.57**
Average raw reads/ library	**49,720,924 ± 9,663,141**
Average % of clean reads/ library	**97.87 ± 0.97**
% of Quality score 20/ library	**97.73 ± 0.15**
% of Quality score 30/ library	**93.91 ± 0.38**
% of GC content/ library	**51.29 ± 2.85**

The standard deviation value for each parameter is indicated for each sample after the symbol ±

## Data Availability

All the raw fastq files from single-cell and bulk RNA sequencing used in this study supporting the conclusions of this article, are deposited in the Sequence Read Archive of the National Center for Biotechnology Information (NCBI) under the BioProject accession number PRJNA1312101(https://www.ncbi.nlm.nih.gov/bioproject/?term=PRJNA1312101). All the scripts used in the data analysis supporting the conclusions of this article are deposited in the Zenodo repository at https://doi.org/10.5281/zenodo.18224827. The hemocyte transcriptome annotation is available at the following website: https://proj-bip-prodpublicread.s3.amazonaws.com/transcriptome/An_gambiae_hemocytes_2022/AgHemocytes.zip. The dataset and the analysis steps related to single-cell and bulk mRNA sequencing supporting the conclusions of this article are also included in the referenced article [16].

## Abbreviations

scRNA-seq: single-cell RNA sequencing
mRNA: messenger RNA
rRNA: ribosomal RNA
RNA-seq: RNA sequencing
An. gambiae: Anopheles gambiae
NaOH: Sodium hydroxide
EDTA: Ethylenediaminetetraacetic acid
NaCl: Sodium chloride
BSA: Bovine Serum Albumin
SPRI: Solid-Phase Reversible Immobilization
qPCR: quantitative Polymerase Chain reaction
7AAD: 7-Amino-Actinomycin D
cDNA: complementary DNA
bp: base pair
ng/µL: nanogram per microliter
µm: micrometer or micron
ng: nanogram
FASTA: Fast Alignment
GTF: Gene Transfer Format
PEST: Pink Eye Standard
UMI: Unique Molecular Identifier
RIN: RNA Integrity Number
Q20/30: Quality Score of 20/30
FACS: Fluorescence-Activated Cell Sorting
NDC: National Drug Code
